# A Caucasian Australian presenting with human T-lymphotropic virus type I associated myelopathy: a case report

**DOI:** 10.1186/1752-1947-8-382

**Published:** 2014-11-23

**Authors:** Matthew Faull, Peter K Panegyres

**Affiliations:** 1 Neurodegenerative Disorders Research Pty Ltd, 4 Lawrence Avenue, West Perth 6005, Western Australia, Australia; 2 Neurology Unit, Joondalup Health Campus, Suite 102, Specialists Medical Centre, Shenton Avenue, Joondalup 6027, Western Australia, Australia

**Keywords:** Aboriginal Australians, Human T-lymphotropic virus type I, Indigenous populations, Myelopathy

## Abstract

**Introduction:**

We report the first known case of human T-lymphotropic virus type I associated myelopathy/tropical spastic paraparesis in an Australian Caucasian, a disease reported in Aboriginal and immigrant populations where the virus is often endemic.

**Case presentation:**

A 41-year-old Caucasian Australian man had a 3-year background of progressive functional decline from a myelopathy with spastic paraparesis and sphincteric dysfunction.

**Conclusions:**

Although studies have shown a very low prevalence of human T-lymphotropic virus type I in the greater Australian population, increased focus on Aboriginal health, and the expanding diversity and integration of the Australian population means that presentation of human T-lymphotropic virus type I-associated disease is likely to increase.

## Introduction

Human T-lymphotropic virus type I (HTLV-1) associated myelopathy or tropical spastic paraparesis (HAM/TSP) is characterised by a slowly progressing spastic paraparesis and HTLV-1 antibodies in serum and cerebrospinal fluid (CSF) [1]. It is a myelopathy predominantly affecting the pyramidal tracts [2] and typically presents as motor dysfunction with a variable degree of sensory dysfunction in the lower limbs and often includes sphincter and bladder disturbances [3,4]. HTLV-1 is endemic in Australia’s Aboriginal population with a seroprevalence of up to 18% in some communities [5]. In the general Australian population however, blood screening has shown that HTLV-1 has a very low seroprevalence of 1 in 100,000 [6]. Carriers are for the most part asymptomatic, a small proportion developing adult T-cell leukaemia/lymphoma, uveitis, infectious dermatitis, polymyositis and neurological syndromes of which HAM/TSP is the most common [3]. The lifetime risk of a carrier developing HAM/TSP is approximately 0.25% to 5% [3,6]. It is still not clear as to why only some carriers develop disease [2]. The first documented cases of HAM/TSP in Australia were described in 1993 in a 31-year-old Aboriginal man [4] and a 64-year-old female Seychellois immigrant [7]. The present case is to the best of our knowledge the first case of HAM/TSP to be described in an Australian Caucasian.

## Case presentation

In 2010, a 41-year-old Caucasian Australian man presented with a 2-week history of urinary incontinence and the inability to walk. Difficulty climbing stairs and occasional foot drop had been present for approximately 3 years prior to presentation. He was also found to have *Pseudomonas* pneumonia, chronic sinusitis, and a small scrotal abscess. Since 1995 he had spent an extensive period in an Aboriginal community in the Great Sandy Desert and was in contact for a total of 4 to 5 years with them. HTLV-1 infection is typically endemic in these Aboriginal populations. He was significantly integrated into the community, undertaking initiation practices such as circumcision and distal urethral hypospadic incision and had unprotected sexual relationships with Aboriginal women. In the Aboriginal community he used intravenous amphetamine and occasionally intravenous heroin with shared needles for several years in addition to cannabis and infrequent ecstasy use. He has had a long history of alcohol abuse with periods of proactive abstinence.

An examination revealed right foot drop, bilateral leg weakness, some quadriceps wasting, bilateral hypertonicity of his lower limbs with sensory impairment reaching his upper thoracic region, and bilateral tingling and numbness in his upper limbs. Urinary incontinence was found to be due to neurogenic bladder dysfunction. Nerve conduction studies ruled out peripheral neuropathy and myopathy. His cranial nerve function was normal. His spinal cord magnetic resonance imaging (MRI) was normal, showing no evidence of cord lesions or compression. Brain MRI showed nonspecific white matter foci related to perivascular spaces and no signs of demyelination.

HTLV-1 antibodies were detected in his serum and CSF. Polymerase chain reaction was positive for HTLV-1 DNA and negative for HTLV-2 DNA in both whole blood and CSF. CSF analysis also revealed oligoclonal bands, elevated protein and a moderate, nonspecific lymphocytosis with no malignant cells or other abnormalities present. Blood investigations revealed a slight normochromic normocytic anaemia, elevated erythrocyte sedimentation rate (ESR; 53 to 108mm/hour over hospital stay) and rouleaux formation, all supporting chronic disease. His red-cell thiamine was normal. His estimated glomerular filtration rate was normal; liver function tests showed elevated gamma-glutamyltransferase and alanine aminotransferase with a normal blood ammonia level. Melioidosis, *Rickettsia*, human immunodeficiency virus, hepatitis B, hepatitis C, *Brucella*, *Strongyloides*, Venereal Disease Research Laboratory test (syphilis) serology were all negative, as was *Mycobacteria* microscopy and culture. Increased polyclonal production of immunoglobulin light chains with normal free light chain ratio was present. Flow cytometry of whole blood revealed expansion of CD4+ lymphocytes with a normal CD4+/CD8+ ratio. Predominant left-sided, chronic sinus disease was detected on computed tomography (CT). His sputum culture produced moderate *Pseudomonas* growth. A chest X-ray in addition to chest, abdominal and pelvis CT were normal apart from a few prominent retroperitoneal lymph nodes of less than 8mm.

On presentation he was started on high dose methylprednisolone (500mg per day for 5 days), resulting in a marked improvement in functionality. His function continued to improve to the point where he could walk into the consultation room on a single elbow crutch. Oxybutynin therapy was initiated to control bladder overactivity and his constipation treated with Coloxyl® (docusate sodium). Six months after initial presentation he presented with complaints of spasticity hindering functionality and sleep. MRI findings at this point again showed no abnormalities. Baclofen was prescribed reducing spasticity and improving functionality. His condition slowly improved until he was walking independently with an ataxic gait and self-catheterising once per day. His myelopathy appeared to have stabilised. On the most recent examination in February 2014, progressive improvement of his myelopathy was noted. He was able to walk 50m without a crutch. Neurological examination revealed pathologically brisk reflexes (3+ in the knees and ankles), plantar flexion, nine beats of ankle clonus on right and six on left. Oxybutynin for bladder control was ceased but he continued to self-catheterise once per day. In addition to baclofen 10mg twice per day, he received, amitriptyline to help him sleep and reduce cramps, as well as Coloxyl® to manage his constipation. He now lives relatively independently.

## Discussion

The slowly progressive neurological symptoms, positive CSF and serum HTLV-1 serology, oligoclonal bands in CSF, elevated ESR lack of radiological signs and other negative findings including negative radiological, serological findings supported the diagnosis of HAM/TSP in this patient. Infection was most likely sexually transmitted or contracted via shared intravenous needles in an Aboriginal community were HTLV-1 infection is endemic. His chronic poor health and chronic alcohol and substance abuse may have impacted on immune-competency and increased his susceptibility to HTLV-1 infection and the development of HAM/TSP.

He had a history of heavy alcohol consumption and monthly intravenous drug usage over many years including amphetamines, cannabis, heroin and ecstasy; he frequently shared needles. He did not have obvious cognitive impairment, encephalopathy or other neuropsychiatric manifestations which might be associated with HTLV-1 neurological manifestations [8].

His brain and spinal cord MRI scans did not reveal diagnostic abnormalities. In one study, spinal cord T2-weighted imaging abnormalities were identified in 3 out of 21 patients with HTLV-1 myelopathy and 11 out of 21 showed nonspecific T2-weighted brain abnormalities [9]; one patient had diffuse transient oedema of the entire spinal cord. These authors have also found poor correlation between clinical observations and MRI findings with cervical demyelination in 3 out of 28 patients, cervical atrophy in 1 out of 28 and enhancing lesions in 1 out of 28; spinal cord lesions correlating with active CSF inflammation. Most patients with HTLV-1 myelopathy and severe neurological disability (Expanded Disability Status Scale ≅6) do not have spinal MRI abnormalities and possess nonspecific brain MRI white matter lesions, as in our patient [10].

The 5-day course of high dose methylprednisolone on initial presentation had a good effect in curtailing the progressive myelopathy and improving symptoms. Although interferon-α therapy has been found to be effective in the treatment of HAM/TSP [11], it was considered impractical due to cost, logistics, the good response of the patient to corticosteroids and his stabilising myelopathy. Cyclosporin is another option, which has shown very promising effects in treating HAM/TSP [12]. Use of cyclosporin was considered inappropriate due to coexistent chest and sinus infection in our patient.

## Conclusions

This case highlights the need to consider HTLV-1 infection in the presentation of myelopathy in patients who have been within areas of Australia or overseas where HTLV-1 infection is endemic. Although blood screening studies have shown a very low prevalence of HTLV-1 in the greater Australian population [6], increased focus on Aboriginal health, and the expanding diversity and integration of the Australian population means that presentation of HTLV-1-associated disease is likely to increase.

## Consent

Written informed consent was obtained from the patient for publication of this case report. A copy of the written consent is available for review by the Editor-in-Chief of this journal.

## Competing interests

The authors declare that they have no competing interests.

## Authors’ contributions

MF analysed and interpreted the patient data, and was a major contributor in writing the manuscript. PKP managed the patient, made the diagnosis, edited and revised the manuscript. Both authors read and approved the final manuscript.

## References

[B1] Nakagawa M Nakahara K Maruyama Y Kawabata M Higuchi I Kubota H Izumo S Arimura K Osame M Therapeutic trials in 200 patients with HTLV-I-associated myelopathy/tropical spastic paraparesis J Neurovirol 1996 2 345 355 10.3109/13550289609146899 8912211

[B2] Nakamura T Nishiura Y Eguchi K Therapeutic strategies in HTLV-I-associated myelopathy/tropical spastic paraparesis (HAM/TSP) Cent Nerv Syst Agents Med Chem 2009 9 137 149 10.2174/187152409788452090 20021347

[B3] Araújo A Lima MA Silva MT Human T-lymphotropic virus 1 neurologic disease Curr Treat Options Neurol 2008 10 193 200 10.1007/s11940-008-0021-1 18579023

[B4] Rajabalendaran N Burns R Mollison LC Blessing W Kirubakaran MG Lindschau P Tropical spastic paraparesis in an aborigine Med J Aust 1993 159 28 29 8316109 10.5694/j.1326-5377.1993.tb137700.x

[B5] May JT Stent G Schnagl RD Antibody to human T-cell lymphotropic virus type I in Australian aborigines Med J Aust 1988 149 104 10.5694/j.1326-5377.1988.tb120516.x 2839756

[B6] Whyte GS Is screening of Australian blood donors for HTLV-I necessary? Med J Aust 1997 166 478 481 9152342 10.5694/j.1326-5377.1997.tb123220.x

[B7] Thyagarajan D Bastian I Stark RJ Day BJ Dax EM Gilligan BS HTLV-I associated myelopathy in a Seychellois immigrant Med J Aust 1993 159 29 31 8316110 10.5694/j.1326-5377.1993.tb137701.x

[B8] Cooper SA Schim van der Leoff M Taylor GP The neurology of HTLV-1 infection Pract Neurol 2009 9 16 26 10.1136/jnnp.2008.167155 19151234

[B9] Bognato F Butman JA Mora CA Gupta S Yamano Y Tasciyan TA Solomon JM Santos WJ Stone RD McFarland HF Jacobson S Conventional magnetic resonance imaging features in patients with tropical spastic paraparesis J NeuroVirology 2005 11 525 534 10.1080/13550280500385039 16338746

[B10] Puccioni-Sohler M Gasparetto E Cabral-Castro MJ Slatter C Vidal CM Cortes RD Rosen BR Mainero C HAM/TSP: association between white matter lesions on magnetic resonance imaging, clinical and cerebrospinal fluid findings Arq Neuropsiquiatr 2012 70 246 251 22510735 10.1590/s0004-282x2012000400004

[B11] Izumo S Goto I Itoyama Y Okajima T Watanabe S Kuroda Y Araki S Mori M Nagataki S Matsukura S Akamine T Nakagawa M Yamamoto I Osame M Interferon-alpha is effective in HTLV-I-associated myelopathy: a multicenter, randomized, double-blind, controlled trial Neurology 1996 46 1016 1021 10.1212/WNL.46.4.1016 8780082

[B12] Martin F Castro H Gabriel C Adonis A Fedina A Harrison L Brodnicki L Demontis MA Babiker AG Weber JN Bangham CR Taylor GP Ciclosporin A proof of concept study in patients with active, progressive HTLV-1 associated myelopathy/tropical spastic paraparesis PLoS Negl Trop Dis 2012 6 e1675 10.1371/journal.pntd.0001675 22720101 PMC3373656

